# Synthesis of *cis*- and *trans*-3-Aminocyclohexanols by Reduction of β-Enaminoketones

**DOI:** 10.3390/molecules17010151

**Published:** 2011-12-27

**Authors:** Iris Montoya Balbás, Blanca Eda Domínguez Mendoza, Mario Fernández-Zertuche, Mario Ordoñez, Irma Linzaga-Elizalde

**Affiliations:** Centro de Investigaciones Químicas, Universidad Autónoma del Estado de Morelos, Av. Universidad 1001, Cuernavaca, Mor., CP 62209, Mexico

**Keywords:** 1,3-amino alcohols, 3-aminocyclohexanols, β-enaminoketones, reduction of β-enaminoketones

## Abstract

We describe a protocol developed for the preparation of β-enaminoketones derived from 1,3-cyclohexanediones, and their subsequent reduction by sodium in THF-isopropyl alcohol to afford *cis*- and *trans*-3-aminocyclohexanols.

## 1. Introduction

Amino alcohols are of great interest because of their biological and structural importance. For example, acyclic 1,3-amino alcohols are key structural components of numerous natural products [1,2,3,4,5,6], potent drugs [7,8], and components of numerous medicinal compounds such as HIV-protease inhibitors [9], *μ*-opioid receptor antagonists [10], potent antibiotic negamycin [11,12,13], serotonin reuptake inhibitor, and antidepressants [14]. Additionally, 1,3-amino alcohols are useful chiral building blocks in asymmetric synthesis functioning as chiral ligands and auxiliaries [15,16,17,18,19,20,21,22,23]. Despite their prevalence and the importance of acyclic 1,3-amino alcohols [24,25,26,27], there are only a few synthetic methods reported in the literature to access to this important class of compounds [28,29,30,31], and even fewer reports exist regarding the synthesis of 1,3-aminocyclohexanols [32,33,34]. We wish to report herein our results on the reduction of β-enaminoketones, leading to the synthesis of both *cis*- and *trans*-3-aminocyclohexanols.

## 2. Results and Discussion

**Scheme 1:**
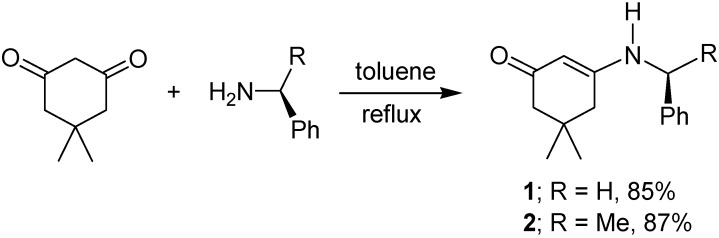
Preparation of β-enaminoketones **1** and **2**.

**Figure 1:**
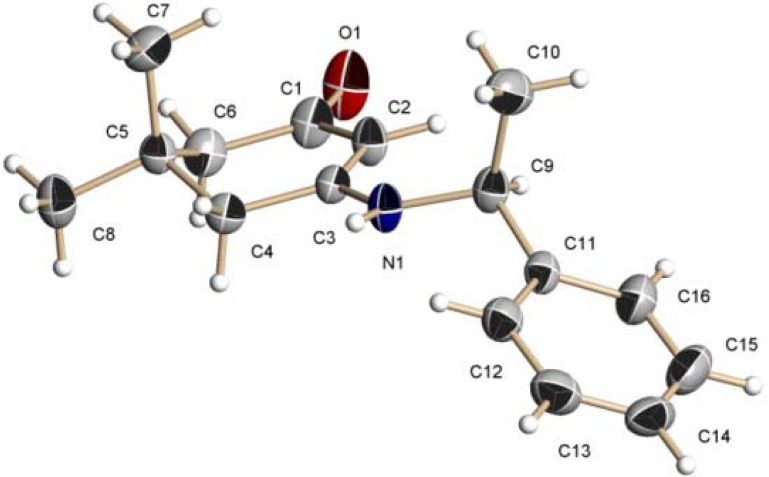
X-Ray structure of β-enaminoketone **2**.

Our method starts with the condensation reaction of 4,4-dimethyl-1,3-cyclohexanedione with either benzylamine or (*S*)-α-methylbenzylamine in toluene at reflux, conditions that lead to the β-enaminoketones **1** and **2** in 85 and 87% yield, respectively (Scheme 1) [35,36]. Both products were fully characterized by NMR spectroscopy and the stereochemistry was corroborated by their X-ray crystal structure [37] (Figure 1).

**Scheme 2:**
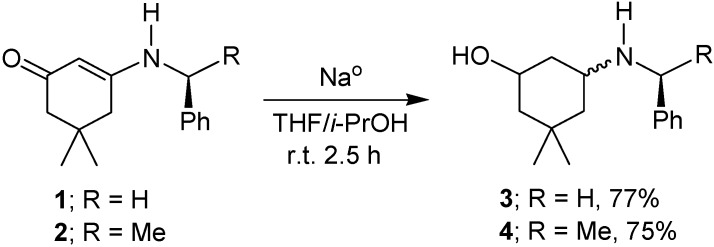
Reduction of β-enaminoketones **1** and **2**.

In a subsequent step, the reduction of β-enaminoketones **1** and **2** was carried out following a procedure described in the literature [38,39,40,41,42,43,44,45]. Thus, the reaction of **1** and **2** with sodium in a mixture of THF/isopropyl alcohol at room temperature afforded the corresponding diasteromeric mixture of amino alcohols **3** and **4** in 77 and 75% yield, respectively (Scheme 2).

A percolation of the reaction mixture followed by GC-MS analysis using a cyclosil-B chiral column revealed the presence of four major stereoisomers in identical ratio for compound **3** and two stereoisomers for compound **4** (*cis* and *trans* in 89:11 ratio). The diastereoisomeric separation of **3** was not attempted; however, column chromatography separation of **4** afforded the *cis*-**4** and *trans*-**4** in 69 and 6% yield, respectively.

**Figure 2:**
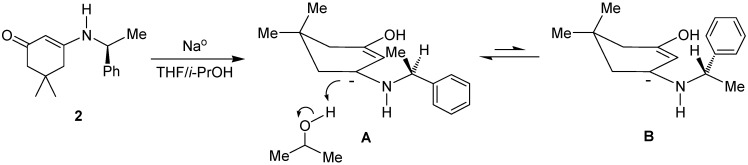
Plausible explanation of the diastereoselectivity in the reduction of **2**.

Considering the X-ray structure of β-enaminoketone **2**, a reasonable explanation of the high *cis*:*trans* diastereoselectivity in its reduction step can be explained assuming that the allyl anion **A** obtained by successive electron-transfers from the sodium to the conjugate system of enaminone, is the more stable conformation, because it avoids the interaction of C-10 or Ph with C2-H observed in conformation **B**. Thus, protonation with isopropyl alcohol of the corresponding allyl anion in the conformation **A** takes place selectively from the bottom-face, since the top-face is hindered by the methyl group (Figure 2).

**Figure 3:**
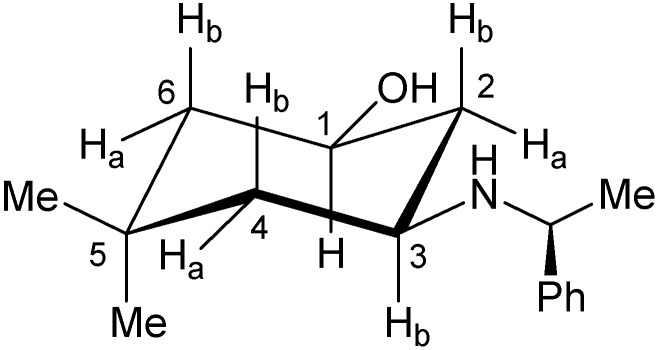
Conformation of the compound *cis*-**4**.

Additionally, structural elucidation for *cis*-**4** and *trans*-**4** was accomplished through ^1^H- and ^13^C-NMR, as well as 2D NMR spectra like COSY, HSQC and NOESY. Spectra data for *cis*-**4** and *trans*-**4** are shown in Table 1. In the ^1^H-NMR spectra of compound *cis*-**4**, protons H_1_ and H_3_ exhibit a triplet of triplets multiplicity, with coupling constants of 11.2, 4.8 Hz and 11.6, 4.0 Hz, respectively. Analysis of these coupling constants confirms the axial disposition of both protons establishing then an equatorial distribution of the OH and NHR groups. Additionally, proton H_2b_ presents a quadruple signal (*J* = 11.6 Hz) which determines its axial position whereas H_2a_ is occupies an equatorial position. The multiplicity of H_4a_ (ddt) shows three couplings constants ^2^*J* = 12.8 Hz, ^3^*J_ec/ax_* = 3.6 Hz and *^4^J_H4a/H6a_* = 2 Hz, this scalar coupling establishes that H_1_ and H_3_ occupy axial positions (Figure 3).

**Table 1:** ^1^H And^13^C-NMR chemical shifts for the compounds *cis*-**4** and *trans*-**4**.

*cis*-4				*trans*-4	
Proton	^1^H *δ*(ppm), *J* (Hz)	Carbon	^13^C *δ* (ppm)	^1^H *δ*(ppm), *J* (Hz)	^13^C *δ* (ppm)
H_1_	3.65 (tt, *J* = 11.2, 4.8, 1H)	C_1_	66.8	3.64 (tt, *J* = 10.8, 4.4, 1H)	67.1
H_2a_	2.13 (m, *J*_gem_ = 11.6, 1H)	C_2_	43.3	2.35 (dddd, *J* = 11.6, 5.6, 4.2, 1H)	42.6
H_2b_	1.07 (q, *J* = 11.6, 1H)			0.94 (bq, *J* = 10.2, 1H)	
H_3_	2.53 (tt, *J* = 11.6, 4.0, 1H)	C_3_	49.5	2.59 (tt, *J* = 11.6, 4.0, 1H)	49.3
H_4a_	1.70 (ddt, *J* = 12.8, 3.6, 2.0, 1H)	C_4_	44.7	1.50 (m, 1H)	46.5
H_4b_	0.97 (t, *J* = 12.0, 1H)			0.99 (t, *J* = 12.0, 1H)	
H_5_	- -	C_5_	31.8	- -	31.7
H_6a_	1.63 (ddt, *J* = 12.4, 4.0, 2.0, 1H)	C_6_	48.1	1.63 (ddt, *J* = 12.4, 4.0, 2.0, 1H)	48.4
H_6b_	0.97 (t, *J* = 11.8, 1H)			1.04 (bq, *J* = 12.0, 1H)	
H_7_	0.97 (s, 3H)	C_7_	33.3	0.93 (s, 3H)	33.2
H_8_	0.70 (s, 3H)	C_8_	26.0	0.75 (s, 3H)	26.2
H_9_	4.00 (q, *J* = 6.4, 1H)	C_9_	55.1	4.03 (q, *J* = 6.8, 1H)	54.8
H_10_	1.42 (d, *J* = 6.4, 3H)	C_10_	24.3	1.40 (d, *J* = 6.8, 3H)	24.9
C_6_H_5_	7.30–7.38 (m, 5H)	C_ipso_	144.3,	7.32–7.35 (m, 5H)	145.4, 128.7, 127.1, 126.7
		C_meta_	128.7,		
		C_ortho_	127.3,		
		C_para_	126.8		
NH, OH	2.37 (bs, 2H)		- -	2.01 (bs, 2H)	- -

**Figure 4:**
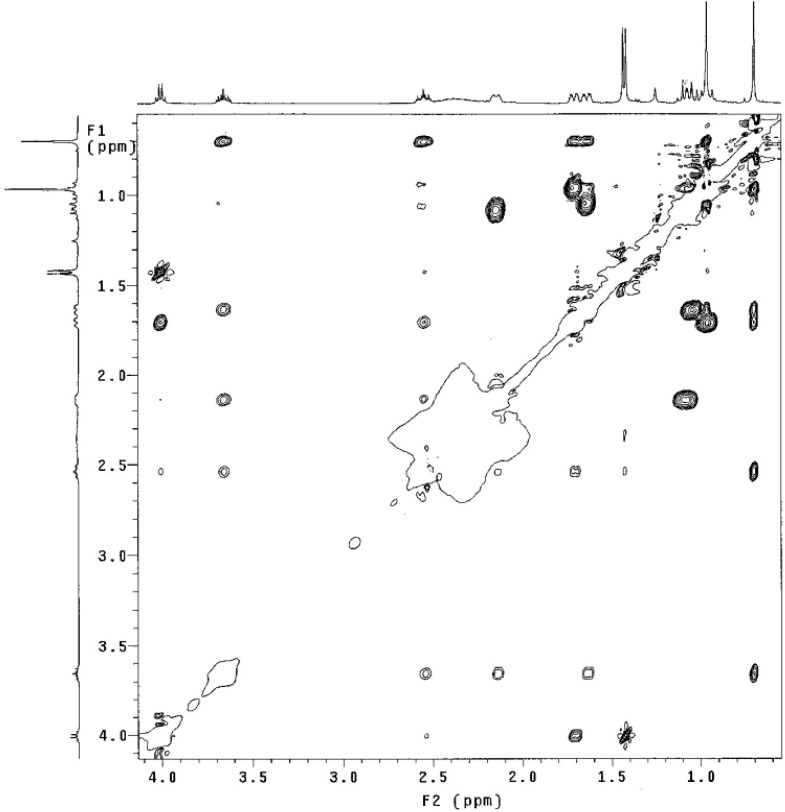
NOESY experiments for *cis*-**4** (CDCl_3_, 400 MHz).

The coupling pattern shown by compound *cis*-**4** establishes a *syn* diequatorial distribution of the OH and NHR groups. A NOESY experiment (Figure 4) carried out on this compound, shows that H_1_ interacts with H_3_ and both protons are close to the equatorial H_2a_. In addition, H_2b_, H_6b_ and H_4b_ show dipolar couplings confirming the analysis of the coupling constants described previously.

**Figure 5:**
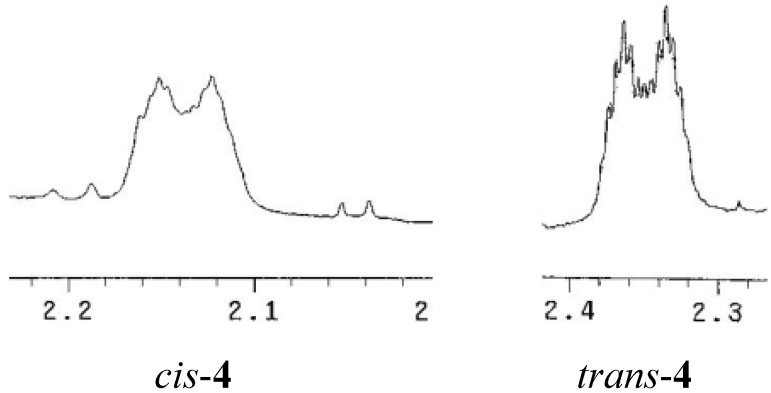
Multiplicity of the protons H_2*ec*_ for compounds *cis*-**4** and *trans*-**4**.

The ^1^H-NMR spectra of the compound *trans*-**4** displays similar data to those observed for the *cis*-**4 **stereoisomer, the main difference being the chemical shift for protons H_2a_, H_2b_, and H_4a_. On the other hand, its ^13^C-NMR data shows that C_4_ is shifted downfield by 2.0 ppm. This can be attributed to a lesser ring strain around this atom. In addition, the coupling pattern for proton H_2a_ is different due to dihedral angles variations (Figure 5).

**Figure 6:**
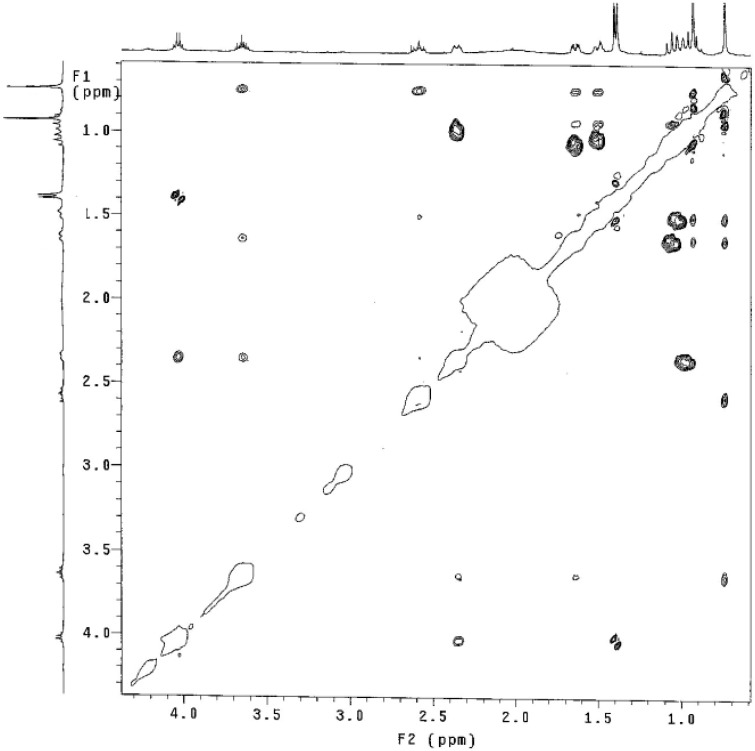
NOESY experiment for compound *trans*-**4** (CDCl_3_, 400 MHz).

Compound *trans*-**4** shows a triplet of triplets for the H_1_ and H_3_ protons (*J* = 11.8, 4.4 Hz and *J* = 11.6, 4.0 Hz respectively), which are similar to those observed for *cis*-**4**. However, in the NOESY experiment (Figure 6) these two protons do not interact spatially, suggesting an *anti*-arrangement of the hydroxyl and amino groups.

**Figure 7:**
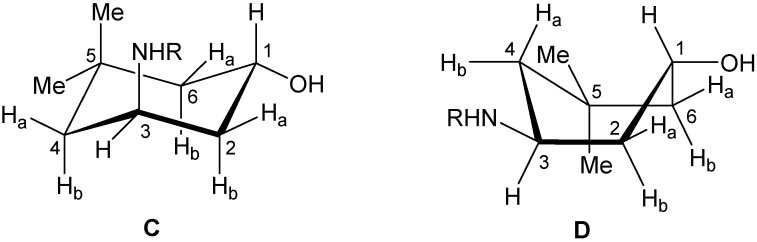
Proposed conformations for the compound *trans*-**4** in solution.

In order to establish the relative configuration at C-1 and C-3 of compound **4**, we also carried out a NOESY experiment (Figure 7). If a chair conformation is considered for compound *trans*-**4** (A), the fact that H_3_ has a dihedral angle below 60° with respect to H_2a_, H_2b_, H_4a_ and H_4b_, would generate coupling constants with magnitude around ~3–5 Hz according to the Karplus rule, however, this is not observed in the spectrum of this compound.

These experimental data thus confirm that the compound *trans*-**4** does not adopt a chair conformation as its isomer *cis*-**4** does. Therefore, we carried out an additional NOESY experiment in order to establish the relative configurations at C-1 and C-3, analyzing the coupling constants and spatial interactions of the two possible conformations **C** and **D** (Table 2). 

**Table 2:** NOE interactions for H_1_, H_3_, H_4eq_ and H_6eq_.

Proton	*cis*-4	*trans*-4
1	3, 2*_eq_*, 4*_eq_*, Me_upfield_	2*_eq_*, 6*_eq_*
3	1, 2*_eq_*, 4*_eq_*, 7ax	Me_upfield_, 4*_eq_*
6*_eq_*	- -	6*_ax_*, Me_downfield_
4*_eq_*	- -	Me-7, Me-8

In conformation **D**, proton H_3_ shows a dihedral angle larger than 120° with H_2a_ and H_4a_, this spatial arrangement exhibits coupling constants of ~12.0 Hz, and the coupling with H_2b_ and H_4b_ of 4.0 Hz. On the other hand the NOESY experiment shows the spatial proximity of H_4b_ to both methyl groups at C_5_, and of proton H_1_ to both H_2a_ and H_6a_. In addition the fact that proton H_3_ shows a proximity to H_4b_, suggests a boat conformation for compound *trans*-**4**. The shielding of H_4a_ is caused by the proximity of the NHR substituent, the torsional effect and the steric hindrance of the boat conformation explains the variation of the chemical shift of C_4_ in comparison to that of compound *cis*-**4**.

## 3. Experimental

Reagents were obtained from commercial suppliers and were used without further purification. Melting points were determined in a Fischer-Johns apparatus and are uncorrected. NMR studies were carried out with a Varian Gemini 200 and Varian Inova 400 instruments using TMS as a standard (^1^H, ^13^C). Chemical shifts are stated in parts per million. IR spectra have been recorded on a Bruker Vector 22 FT spectrophotometer. The diastereoisomeric composition were determined by GC-MS on the HP 5989A, Cyclosil-B column, 30 m, 0.25 mm (ID), 0.25 μm (film), transfer line 220 °C, injection 220 °C, and HRMS in Jeol JMS 700 equipment. X-ray diffraction studies were performed on a Bruker-APEX diffractometer with a CCD area detector at 100 K (λ_Mo__Kα_ = 0.71073 A, monochromator: graphite). Specific rotations were measured in a Perkin-Elmer 341 polarimeter at room temperature and λ = 589 nm.

### 3.1. General Experimental Procedures

*5,5-Dimethyl-3-benzylaminocyclohexen-2-one* (**1**). A solution of 4,4-dimethyl-1,3-cyclohexanedione (1.0 g, 7.13 mmole) and benzylamine (0.86 mL, 7.84 mmole) was refluxed in toluene (30 mL) for 3 h, while the water formed was azeotropically removed using a Dean-Stark trap. After this time, the solvent was removed under reduced pressure and the resulting yellow solid was purified by recrystallization (CH_2_Cl_2_/hexane) affording **1** (1.39 g, 85%), mp = 122–125 °C. IR (film CH_2_Cl_2_, cm^−1^): 3,252, 3,062, 1,800, 1,545. ^1^H-NMR (400 MHz, CDCl_3_): δ 1.05 (s, 6H), 2.14 (s, 2H), 2.25 (s, 2H), 4.23 (d, *J* = 10.8 Hz, 2H), 5.14 (s, 1H), 5.77 (bs, 1H), 7.30 (m, 5H). ^13^C-NMR (100 MHz, CDCl_3_): δ 28.5(2), 33.0, 43.5, 47.3, 50.2, 95.9, 127.6(2), 127.9(2), 128.9, 136.9, 163.5, 196.7. HRMS CI^+^ calcd. for C_15_H_20_NO (M^+^+1): 230.1545. Found: 230.1538.

*(S)-5,5-Dimethyl-3-(α-methylbenzylamino)cyclohexen-2-one* (**2**). A solution of 4,4-dimethyl-1,3-cyclohexanedione (1.0 g, 7.13 mmole) and (*S*)-α-methylbenzylamine (1.0 mL, 7.84 mmole) was refluxed in toluene (30 mL) during 3.5 h, while the water formed was removed azeotropically using a Dean-Stark trap. After this time, the solvent was removed and the yellow solid obtained was purified by crystallization (CH_2_Cl_2_/hexane) to give compound **2** (1.51 g, 87%), mp = 135–137 °C. [α]_D_ = −243.26 (*c* = 1, CHCl_3_). IR (KBr, cm^−1^): 3,270, 3,059, 1,750, 1,542 cm^−1^. ^1^H-NMR (200 MHz, CDCl_3_): δ 1.01 (s, 3H), 1.06 (s, 3H), 1.47 (d, *J* = 6.6 Hz, 3H), 2.12 (s, 2H), 2.23 (s, 2H), 4.47 (q, *J* = 6.6 Hz, 1H), 4.97 (s, 1H), 5.62 (d, *J* = 6 Hz, 1H), 7.26 (m, 5H). ^13^C-NMR (50 MHz, CDCl_3_): δ 23.6, 28.5(2), 33.1, 43.6, 50.1, 53.0, 97.1, 125.7(2), 127.5, 128.9(2), 142.9, 162.5, 196.5. HRMS CI^+^ calcd. for C_16_H_22_NO (M^+^+1): 244.1701, found: 244.1695.

### 3.2. General Procedure for the Reduction of β-Enaminoketones ***1*** and ***2***

The β-enaminoketones (2.0 mmol) were dissolved in a mixture of isopropyl alcohol (2 mL) and THF (5 mL). The resulting solution was treated with an excess of small pieces of metallic sodium (0.27 g, 12.0 g-atoms) and stirred from 0 °C to room temperature until the reaction was complete (TLC). After removal of the unreacted sodium, the reaction mixture was poured into a saturated aqueous solution of NH_4_Cl and extracted with AcOEt. The organic layers were combined, dried over Na_2_SO_4_ filtered and evaporated under reduced pressure. The resulting materials were submitted to an initial percolation and then were submitted to HPLC-MS analysis. The materials were separated by column chromatography (silica gel, 230–400) eluting with 65:25:10 proportions of hexane/ethyl acetate/isopropyl alcohol or 95:5, CH_2_Cl_2_/CH_3_OH.

*5,5-Dimethyl-3-benzylaminocyclohexanols* (**3a**,**b**). Compound **3a**: (97 mg, 48%). ^1^H-NMR (400 MHz, CDCl_3_): δ 0.85 (s, 3H), 0.99 (s, 3H), 1.09 (m, 3H), 1.67 (m, 2H), 2.31(m, *J_gem_* = 11.6 Hz, 1H), 2.79 (tt, *J* = 11.6, 4 Hz, 1H), 2.95 (bs, 2H), 3.75 (tt, *J* = 11.2 4.4 Hz, 1H), 3.83 (d, *J* = 12.8 Hz, 1H), 3.85 (d, *J* = 13.2 Hz, 1H), 7.3 (m, 5H). ^13^C-NMR (50 MHz, CDCl_3_): δ 26.3, 31.8, 33.3, 42.7, 45.3, 48.2, 50.9, 51.7, 66.6, 127.2, 128.4, 128.6, 139.7. MS, CI^+^ (M^+^+1): 234, 216, 162, 91. HRMS CI^+^ calcd. for C_15_H_24_NO (M^+^+1): 234.1858, found 234.1891. Compound **3b**: (59 mg, 29%). ^1^H-NMR (400 MHz, CDCl_3_): δ 0.89, (s, 3H), 0.99 (s, 3H), 1.04 (m, 3H), 1.6 (bs, 2H), 1.65 (ddt, *J* = 12.8, 4, 2 Hz, 1H), 1.70 (ddt, *J* = 12.8, 4, 2 Hz, 1H), 2.29 (dddd, *J* = 11.6, 4, 2 Hz, 1H), 2.76 (tt, *J* = 11.2, 4 Hz, 1H), 3.79 (m, 1H), 3.8 (d, *J* = 12.8 Hz, 1H), 3.84 (d, *J* = 12.8 Hz, 1H), 7.3 (m, 5H). ^13^C-NMR (100 MHz, CDCl_3_): δ 25.9, 31.8, 33.1, 41.3, 43.2, 47.8, 49.7, 51.7, 66.5, 128.2, 128.9, 129.3, 136.3. MS, CI^+^ (M^+^+1): 234, 216, 174, 162, 108, 106, 91. HRMS calcd. for C_15_H_24_NO (M^+^+1): 234.1858, found 234.1852.

*5,5-Dimethyl-3-[(S)-α-methylbenzylamino]cyclohexanol* (*cis*-**4** and *trans*-**4**). Compound *cis*-**4**: (352 mg, 69%), [α]_D_ = −48 (*c* = 3.26, CHCl_3_). IR (KBr, cm^−1^): 3439, 3257, 3028, 1646. ^1^H-NMR (400 MHz, CDCl_3_): δ 0.70 (s, 3H), 0.97 (s, 3H), 0.97 (t, *J* = 11.8 Hz, 1H), 0.97 (t, *J* = 12 Hz, 1H), 1.07 (q, *J* = 11.6 Hz, 1H), 1.42 (d, *J* = 6.4 Hz, 3H), 1.63 (ddt, *J* = 12.4, 4.2 Hz, 1H), 1,70 (ddt, *J* = 12.8, 3.6, 2.0 Hz, 1H), 2.13 (m, *J_gem_* = 11.6 Hz, 1H), 2.37 (bs, 2H), 2.53 (tt, *J* = 11.6, 4.0 Hz, 1H), 3.65 (tt, *J* = 11.2, 4.8 Hz, 1H), 4.00 (q, *J* = 6.4 Hz, 1H), 7.30–7.38 (m, 5H). ^13^C-NMR (100 MHz, CDCl_3_): δ 24.3, 26.0, 31.8, 33.3, 43.3, 44.7, 48.1, 49.5, 55.1, 66.8, 126.8, 127.3, 128.7, 144.3. MS CI^+^ (M^+^+1): 248, 247, 232, 230, 176, 105. HRMS CI^+^ calcd. for C_16_H_26_NO (M^+^+1): 248.2014, found 248.2132. Compound *trans*-**4**: (32 mg, 6%) [α]_D_ = −28 (*c* = 0.24, CHCl_3_). IR (KBr, cm^−1^): 3,376, 3,067, 3,029, 1,633. ^1^H-NMR (400 MHz, CDCl_3_): δ 0.75 (s, 3H), 0.93 (s, 3H), 0.94 (bq, *J* = 10.2 Hz, 1H), 0.99 (t, *J* = 12 Hz, 1H), 1.04 (bq, *J* = 12 Hz, 1H), 1.40 (d, *J* = 6.8 Hz, 3H), 1.50 (m, 1H), 1.63 (ddt, *J* = 12.4, 4.2 Hz, 1H), 2.01 (bs, 2H), 2.35 (dddd, *J* = 11.6, 5.6, 4.2 Hz, 1H), 2.59 (tt, *J* = 11.6, 4.1 Hz, 1H), 3.64 (tt, *J* = 10.8, 4.4 Hz, 1H), 4.03 (q, *J* = 6.8 Hz, 1H), 7.32–7.35 (m, 5H). ^13^C-NMR (50 MHz, CDCl_3_): δ 24.9, 26.2, 31.7, 33.2, 42.6, 46.5, 48.4, 49.3, 54.8, 67.1, 126.7, 127.1, 128.7, 145.4. MS CI^+^ (M^+^+1): 248, 247, 232, 230, 176, 105. HRMS CI^+^ calcd. for C_16_H_25_NO (M^+^+1): 248.2014, found 248.2341.

## 4. Conclusions

In conclusion, 1,3-amino alcohols **3** and **4** were obtained as diastereoisomeric mixtures in good yield by reduction of the corresponding β-enaminoketones **1** and **2**, which were analyzed by gas chromatography/mass spectrometry using a chiral column. Two diastereomeric pairs were identified for compound **3** and two diasteromeric pairs, *cis*-**4** and *trans* **4**, for compound **4**. Chromatographic techniques allowed the separation of *cis*-**4** and *trans*-**4**. On the other hand, NMR NOESY experiments enabled us to establish a chair conformation and a *syn*-orientation of the hydroxyl and amino groups for *cis*-**4** and a boat conformation with *anti*-orientation of the hydroxyl and amino groups for *trans*-**4**.
